# Histone deacetylase 2 regulates ULK1 mediated pyroptosis during acute liver failure by the K68 acetylation site

**DOI:** 10.1038/s41419-020-03317-9

**Published:** 2021-01-11

**Authors:** Yao Wang, Qian Chen, Fangzhou Jiao, Chunxia Shi, Maohua Pei, Luwen Wang, Zuojiong Gong

**Affiliations:** Department of Infectious Diseases, Renmin Hospital of Wuhan University, Wuhan, 430060 P.R. China

**Keywords:** Acetylation, Hepatotoxicity

## Abstract

Pyroptosis is a new necrosis pattern of hepatocyte during liver inflammation in acute liver failure (ALF). Histone deacetylase 2 (HDAC2) is associated with several pathological conditions in the liver system. The aim of this study is to investigate whether knockdown or pharmacological inhibition of HDAC2 could reduce the level of pyroptosis in ALF through ULK1-NLRP3-pyroptosis pathway. The role of HDAC2 on ULK1-NLRP3-pyroptosis pathway during ALF was detected in clinical samples. The mechanism was investigated in transfected cells or in ALF mouse model. The RNA-sequencing results revealed that ULK1 was a negative target regulatory molecule by HDAC2. During the process of pyroptosis, the HDAC2 exerted the antagonistic effect with ULK1 by the K68 acetylation site in L02 cells. Then the role of HDAC2 on ULK1-NLRP3-pyroptosis pathway in ALF mouse model was also detected. Moreover, the related molecules to ULK1-NLRP3-pyroptosis pathway were verified different expression in normal health donors and clinical ALF patients. HDAC2 in hepatocytes plays a pivotal role in an ULK1-NLRP3 pathway driven auto-amplification of pyroptosis in ALF. One of the important mechanisms is that inhibition HDAC2 to reduce pyroptosis may be by modulating the K68 lysine site of ULK1.

## Introduction

Acute liver failure (ALF) is caused by metabolic and infectious factors characterized by a large number of deaths of liver cells in a short period of time. Due to the rapid onset and high mortality of ALF, it is currently no breakthrough for the treatment^1^. Studies have shown that patients with ALF accompany by severe intestinal micro-ecological imbalance induced causes intestinal endotoxemia^2^. The intestinal endotoxin can not only induce liver localized non-specific hypersensitivity reaction (Shwartzman reaction), but also stimulate the release of cytokines, like tumor necrosis factor-alpha (TNF-α), interleukin (IL)-1α, IL-6 in liver macrophages^3^. These cytokines act synergistically with innate immune inflammatory system to further produce a natural immune cascade aggravating hepatocyte damage during ALF^4^.

As a new way of cell damage, the cell pyroptosis is characterized by caspase 1 dependence, accompanied by rapid cleavage of the plasma membrane and a large release of pro-inflammatory factors (IL-1β and IL-18)^5^. In the classical process of pyroptosis, a pattern recognition receptor such as nucleotide-binding oligomerization domain-like receptor protein 3 (NLRP3) combines with cysteine protease pro-caspase 1 to form inflammasome. Under the stimulation of inflammatory factors, inflammasome can process pro-caspase 1 into mature caspase 1. It promotes subsequently the secretion of IL-1β and IL-18^5^. During this process, the increased pore size of the plasma membrane is destroyed by the structure of N-terminal gasdermin D (GSDMD) to aggregate on cytomembrane. Then the cells gradually swell until the cells rupture. A large amount of cellular contents, such as IL-1β and IL-18 are released, trigger a strong inflammatory response^6^. In the process of pyroptosis, hepatocytes are activated by damage-associated molecular pattern (DAMPs) and pathogen-associated molecular patterns (PAMPs) to activate nuclear factor-κB, which in turn activates NLRP3, and makes the formation of NLRP3-apoptosis associated speck like protein containing a CARD (ASC) inflammasome. It would further activate pro-caspase 1, promote liver pyroptosis^7,8^.

Studies have demonstrated that autophagy can negatively regulate the activation of inflammasome^9,10^. The autophagy/lysosomal system can down-regulate misfolded polymerized proteins and dysfunctional organelles during the inflammasome, which involved in inflammatory response^10^. As the earliest discovered autophagy gene, unc-51 like kinase 1 (ULK1) can promote autophagy and inhibit the activity of NLRP3 inflammasome^11,12^. Therefore, autophagy-NLRP3- pyroptosis pathway plays an important role in the cell injury and inflammatory response. If ULK1 could be upregulated, the degree of pyroptosis and inflammation would be suppressed.

In our previous study, inhibitor of histone deacetylase 2 (HDAC2), CAY10683 was verified to reduce intestinal epithelial cell apoptosis^13^, protective intestinal mucosal barrier through LPS/TLR4/MyD88 pathway in ALF^14^. Moreover, modulation of HDAC2 offers a protective effect through the mitochondrial apoptosis pathway in ALF^15^. However, it is still unknown the mechanisms of modulations of HDAC2 affecting on the proapoptotic specific and/or antiapoptotic molecules. At present, there is no report on the influence of acetylation on pyroptosis in ALF. This study aimed to determine whether HDAC2 could regulate autophagy-NLRP3-pyroptosis pathway in the process of ALF. The key molecules and their modification sites regulated by HDAC2 in this pathway would be identified by gene sequencing and mass spectrometry.

## Materials and methods

### RNA-sequencing (RNA-seq) library analysis

HeLa cells were cultured in DMEM supplemented with 10% FBS (HyClone, Cat.No.10099) and 1% L-glutamine. The cells were transfected with plasmid to knock-down or overexpress HDAC2. After being transfected for 24 h, total RNA was extracted and then treated with DNAzyme to remove DNA. RNA integrity was further verified by 1.5% Agarose gel electrophoresis. For each sample, 10 μg of total RNA was used for RNA-seq library preparation. Polyadenylated mRNAs were purified and concentrated with oligo(dT)-conjugated magnetic beads (Invitrogen) before being used for directional RNA-seq library preparation. Purified mRNAs were fragmented at 95 °C in fragmentation buffer. Reverse transcription was performed. The cDNAs were purified and amplified. For high-throughput sequencing, the libraries were prepared following the manufacturer’s instructions and applied to Illunima HiSeq X Ten system for 150 nt paired-end sequencing. In the part of analysis, differentially expressed genes (DEG) between groups were analyzed by edgeR software.

### Cell culture and chemical administration

The human liver cell line L02, that was recently authenticated and tested with no mycoplasma contamination, was purchased from Cell Collection Center of Wuhan University (Wuhan, China). The cells were cultured with DMEM medium mixed with 10% FBS in the incubator with in the 37 °C, 5% CO_2_ concentration, saturated humidity environment. TNF-α (Sigma, USA) combined with D-Galactosamine (D-Gal, Sigma, USA) were used to establish in vitro pyroptosis model. Then TNF-α (100 ng/mL) and D-Gal (44 μg/mL) were used to stimulate the cells except for the control group. Inhibitor of histone deacetylase 2 (HDAC2), CAY10683 (120 nM, Sellect, USA) were treated cells after a 24 h incubation. The intervention groups were stimulated with ULK1 inhibitor MRT68921 (2.9 nM, Tocris, USA), pyroptosis inhibitor AC-YVAD-CMK (8 μM, Tocris, USA)^16^, CAY10683 or combined intervention with the above three drugs for 2 h at 37 °C in advance of further treatment. After 2 h, TNF-α and D-Gal were added to the cells for 24 h. AC-YVAD-CMK is a potent and irreversible inhibitor of the inflammatory caspase 1^17^. It is a tetrapeptide sequence based on the target sequence of caspase 1 in pro-IL-1β^17,18^. This drug is described as blocking inflammatory cell death in experimental models^19^, which displays anti-inflammatory and anti-pyroptotic effects^20,21^. MRT68921 inhibited the autophagy-related LC3-II flux that promote by mTOR inhibitor AZD8055. Further research has found that MRT68921 specifically disrupts autophagosome maturation and thus blocks autophagic flux via ULK1 kinase inhibition^22^.

### Plasmid, lentiviral vectors construction, and transfection

The knockdown short hairpin RNA (shRNA) and overexpressed plasmid for HDAC2 were constructed. The shRNA and plasmid were then enveloped by lentiviral vectors (LV, GeneCreate, China). The knockdown interference sequence was 5′-GCAAATACTATGCTGTCAATT-3′. The cloning vectors were transfected into the 293 T cell line, and the viral supernatant was harvested after 48 h (1 × 10^8^ transducing units (TU)/mL). Then, L02 cells were seeded into 24-well plates and transfected with lentivirus vectors. According to the manufacturer’s instructions, the tested multiplicity of infection (MOI) value was 40. After being transfected for 72 h, the corresponding drugs were intervened. The ULK1 overexpression plasmid conducted and subcloned into the pCMV3 vector by HYcell biotechnology (Wuhan, China). Two HA-tagged ULK1 constructs, ULK1- wild type (WT)-HA and ULK1-K686R-HA vector were constructed by GenePharma (Shanghai, China). ULK1-K68R-HA and ULK1-WT-HA mutants were generated by using a QuikChange II site-directed mutagenesis kit (Stratagene) following by the manufacturer’s instructions. For cell transfection, plasmids were mixed with Lipofectamine 2000 (Invitrogen, USA) and transfected into L02 cells according to the manufacturer’s instructions.

### Animal groups

All animal experiments were conducted according to the National Institutes of Health guidelines and were approved by the Institutional Animal Care and Use Committee of Renmin Hospital of Wuhan University. The animal quality certificate was No. 11400700348230. The laboratory animal facility use license number was No. SYXK (Hubei) 2015–0027. The ethical approval for research involving animal number was WDRM (Welfare) 20181018. Meanwhile, the animal experiment followed the ARRIVE guidelines^23^. Sixty-four male specific pathogens free (SPF) C57BL/6 mice (Hubei Animal Experimental Center) weighing 20–25 g and 6–8 weeks old were housed in the animal experiment center in Renmin Hospital of Wuhan University with 12 h light/dark cycle, temperature of 25 ± 2 °C and relative humidity of 50% ± 15%. All mice were provided with food and water ad libitum. All mice were equally divided into 8 groups: normal group, LPS/D-Gal group, LPS/D-Gal + CAY10683 group, LPS/D-Gal + AC-YVAD-CMK group, LPS/D-Gal + MRT68921 group, LPS/D-Gal + MRT68921 + CAY10683 group, LPS/D-Gal + MRT68921 + AC-YVAD-CMK group, LPS/D-Gal + MRT68921 + AC-YVAD-CMK + CAY10683 group. The dose of MRT68921 was 5 mg/kg, and one dose was given intraperitoneally 2 h before modeling. Referring to the reported dose intraperitoneally^24^, AC-YVAD-CMK was administered at a dose of 6 mg/kg, and was administered intraperitoneally 2 h before modeling. The dose of CAY10683 was 3 mg/kg, and one dose was given 2 h before modeling.

### Specimen collection, histological, and biochemical examination

All investigations with human subjects were conducted according to the principles expressed in the Helsinki Declaration. The study was signed with informed consent of patient and agreed with Ethics Committee of Renmin Hospital of Wuhan University. A total of 176 the subjects from Renmin Hospital of Wuhan University were enrolled from March 2018 to March 2019, including 70 healthy donors and 106 liver failure patients. The diagnosis of liver failure complies with the “European Association for the Study of the Liver (EASL) Clinical Practical Guidelines on the management of acute (fulminat) liver failure”^25^. The patients were excluded liver malignant tumors, patients undergoing liver transplantation, pregnant or lactating women. The ALF patient and normal donor liver tissues were provided by the Liver Transplantation Center of Renmin Hospital of Wuhan University. The ethics committee of Renmin Hospital of Wuhan University approved the informed consent of all subjects.

Mice and human fresh liver specimens were fixed in 10% neutral-buffered formalin or 4% glutaraldehyde and then processed for sectioning and staining by standard histological methods. Sections from the liver were stained with HE and evaluated under light microscope (Olympus, Tokyo, Japan). Liver samples and L02 cells were fixed by 4 % glutaraldehyde. After dehydration by ethanol and acetone, epoxy resin was embedded and sliced, and saturated uranium acetate and lead citrate were stained double. The ultrastructure and autophagosome changes of L02 cell and liver tissues were observed by transmission electron microscope. The immunohistochemical detection was performed following by previously described method^26^. The primary antibodies of HDAC2 (1:200; Abcam, USA), ULK1 (1:200; CST, USA), NLRP3 (1:100; CST, USA), GSDMD (1:200; Proteintech, China), caspase 1 (1:200; Abcam, USA), IL-18 (1:200; Proteintech, China), IL-1β (1:200; Proteintech, China) were used to detect the level of autophagy-NLRP3-pyroptosis, respectively. Five fields in every section were randomly counted by ×200 magnification under microscopy. According to the method of Axiotis et al.^27^, immunostaining was semi-quantitatively expressed as follows: percentage of liver cells staining: 0 point, 1–10%; 1 point, 11–25%; 2 points, 26–50%; 3 points, 51–75%; 4 points, 76–100%; and color of immunostaining: 0 point, none; 1 point, light brown; 2 points, brown; 3 points, dark brown. The two indicator scores were multiplied: 0 point, negative (−); 1–4 points, weakly positive (+); 5–8 points, moderately positive (++); 9–12 points, strong positive (+++).

The fresh sera of 281 subjects were collected. Age and sex were recorded. The blood routine examination (including white blood cell count, neutrophil percentage, red blood cell count, hemoglobin concentration, platelet count), blood coagulation index: (activated partial thromboplastin time (APTT), prothrombin activity (PTA), prothrombin time (PT)), liver functions: alanine aminotransfetase (ALT), aspartate transaminase (AST), total bilirubin (TBIL), albumin, and inflammation indicators including procalcitonin (PCT), C-reactive protein (CRP), high-sensitivity C-reactive protein (hs-CRP) were tested by the fully automated Aeroset chemistry analyzer (Abbott Co. Ltd., USA). Serum HDAC2 level was detected according to the protocol of assay kit (BioVision, USA). Serum and cellular supernatant IL-18, IL-1β levels were detected by enzyme-linked immunosorbent assay (ELISA) kits (Elabscience, China).

The pyroptosis kit (Immunochemistry, USA) was used to detect the pyroptosis rate by flow cytometry. Lactate dehydrogenase (LDH) assay kit (Solarbio, China), ALT and AST activity assay kits (Solarbio, China) were used to detect the degree of liver injury. ALT is the most abundant in the liver and only distributed in the cytoplasm. AST is an intracellular functional enzyme, 80% of which is present in the mitochondria of liver cells. ALT and AST are released into the blood after hepatocyte damage. The liver is the only place for serum bilirubin metabolism. Massive necrosis of liver cells, severely impaired liver function, resulting in bilirubin metabolism disorders. It subsequently leads serum TBIL level increased significantly. Therefore, the levels of ALT, AST, and TBIL reflect the degree of hepatocyte damage and necrosis in the process of ALF^28^.

### Western blotting and immunoprecipitation (IP) detection of protein expression

The cells or liver tissues were homogenized by radio immunoprecipitation assay (RIPA) lysis buffer (Beyotime, China) on ice to extract total protein. Protein concentration was determined by bicinchoninic acid (BCA) protein assay reagent assay kit (Beyotime Institute of Biotechnology). Protein lysates (30 μg) were subjected to 12% SDS -PAGE. The proteins were transferred to polyvinylidene fluoride (PVDF) membranes (Millipore, USA). After blocking with 20% non -fat milk at room temperature for 1 h, the membranes were incubated with primary antibodies against HDAC2 (1:1000), ULK1(1:1000), NLRP3 (1:500), pro-caspase 1 + p 10 + p 12 (1:1000), GSDMD (1:1000) IL-18 (1:1000), IL-1β (1:1000) and GAPDH (1:2000) overnight at 4 °C, respectively.

Cells transfected with ULK1-pCMV3 vector and administrated and corresponding drugs were lysed in IP buffer. The lysates were incubated with 5 μg of primary antibody or Ig G (Beyotime, China) for 4 h at 4 °C. 30 μl of Protein A/G PLUS-Agarose (Santa Cruz, USA) was then added. The samples were incubated overnight at 4 °C. The precipitates were washed 5 times with IP buffer. Next, the beads were resuspended in 40 μl of 1.5× loading buffer, boiled for 5 min, and centrifuged at 2500 × rpm for 5 min. The supernatants were collected and subjected to SDS-PAGE by western blotting. ULK1 (1:1000) and acetylated lysine (Ac-Lys, 1:1000, CST, USA) primary antibodies were used in the IP experiment.

All the above bands were incubated by the IRDye800CW secondary antibody (1:10,000, LI-COR, USA) at 37 °C for 1 h. The bands were visualized by the Odyssey Infrared Imaging System (version 3.0, LI-COR Biosciences).

### Immunofluorescence detection for HDAC2, ULK1, and lysosomes in cells

For immunofluorescence, glass slides were placed in 24-well plates and were seeded with 1 × 10^4^ L02 cells/400 μl DMEM per plate. After treatments, the slides were fixed with 4% paraformaldehyde at 37 °C for 30 min, permeabilized with 0.2% Triton X-100 (Beyotime Institute of Biotechnology) at 37 °C for 20 min, blocked with 5% bovine serum albumin (BSA, Beijing Solarbio Science & Technology Co., Ltd.) at 37 °C for 30 min and incubated with primary antibodies against HDAC2 (1:100), ULK1 (1:100) and lysosome (Lyso) red fluorescent probe Lyso-Tracker Red (60 nM, Beyotime, Wuhan, China) at 4 °C overnight, respectively. Slides were then incubated with Cy3 and FITC-labeled fluorescent secondary antibodies (1:100, proteintech, China) at 37 °C for 1 h in dark room. Then the sections were stained with 5 μg/ml 4′,6-diamidino-2-phenylindole (DAPI, Beyotime, Wuhan, China) at 37 °C for 5 min in a dark room. The slides were observed under a laser confocal microscopy at ×600 magnification.

### Identification and analysis of protein IP-mass spectrometry (MS)

Liver tissues and L02 cells were lysed by IP lysis buffer. The samples were digested into peptides by protein enzymatic hydrolysis after IP and western blot experiments. LC-MS/MS analysis was performed using an Easy nLC 1200 chromatography system (Thermo Scientific, USA). The mass spectrometry database retrieval software MaxQuant 1.6.2.0 was used in this study. The Swiss-Protein Database was searched to analyze the data.

### Statistical analysis

The data were processed using SPSS version 18.0 statistical software. Measurement data were expressed as mean ± SEM. *P* < 0.05 was considered statistically significant. The experimental data were assessed by Student’s *t* test, one-way ANOVA, or two-way ANOVA procedure. Tukey’s post hoc tests were performed only if the *F* value in ANOVA achieved the necessary level of statistical significance. Equalization and randomization, were used for each group in both in vivo and in vitro experiments, including mouse allocation, experimental performance, data extraction, and data analysis.

## Results

### The expression of ULK1 gene was negatively correlated with the expression of HDAC2 through bioinformatics analysis

**Fig. 1:**
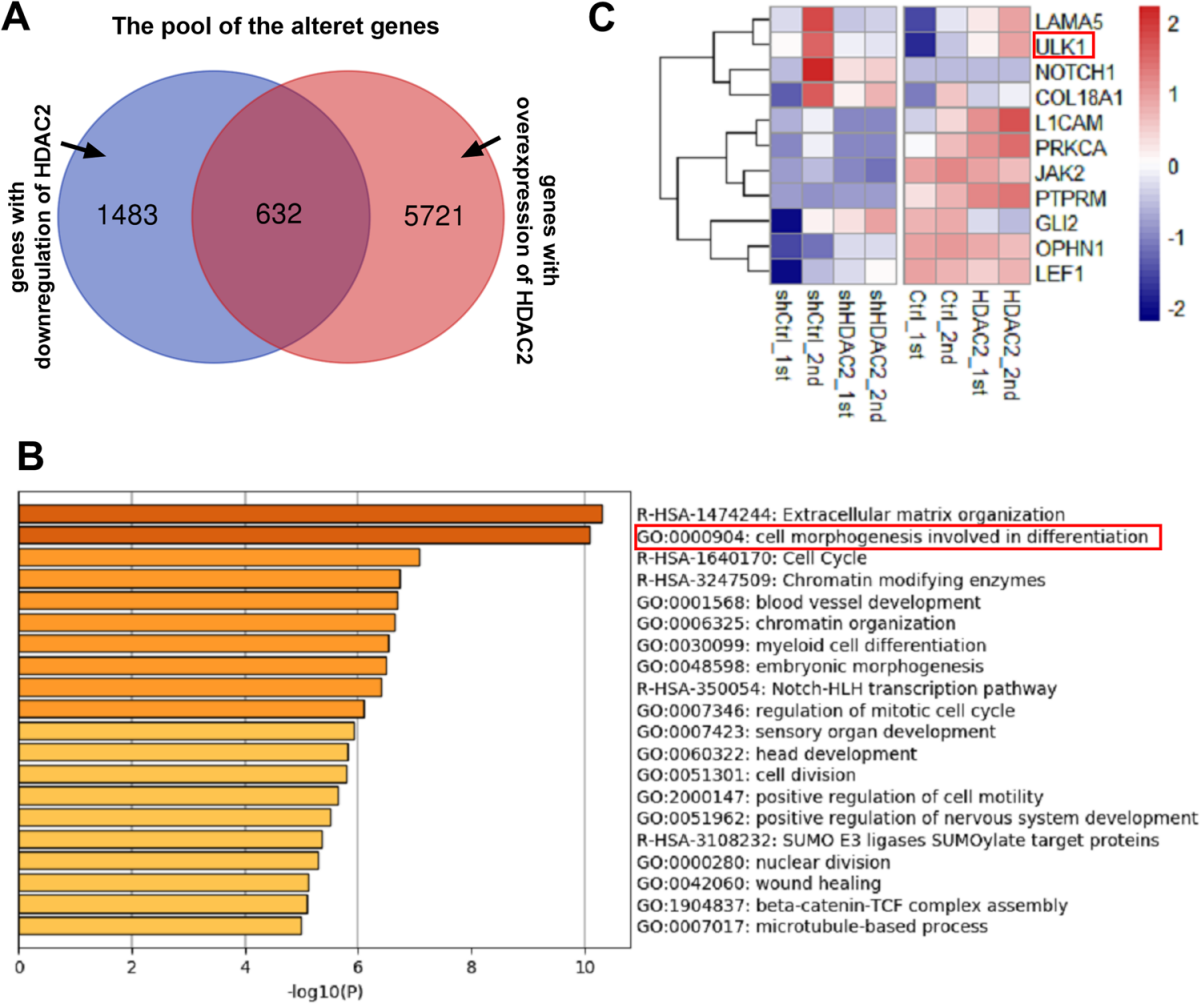
The expression of ULK1 gene being negatively correlated with the expression of HDAC2 through bioinformatics analysis. **A** The bioinformatics analysis for the Venn diagram revealed that 1483 and 5721 genes were differentially altered after knockdown and overexpression of HDAC2. Among these changed genes, there were 636 genes altered in both HDAC2 upregulated or downregulated, respectively. **B** These 636 genes were performed with gene ontology (GO) analysis. There was no gene involved in pyroptosis directly regulated by HDAC2 among the 636 genes. It was found that cell morphogenesis involved in differentiation for the GO Biological Processes was relatively closely related to the characteristic of pyroptosis that the cell expanded until the cell membrane ruptured, leading to the release of its contents. **C** 11 genes with high correlation in the process of cell morphogenesis involved in differentiation were clustered. ULK1 was only autophagy molecule associated the NLRP3-pyroptosis pathway in the 11 genes.

In our previous study, it was found that inhibition of the activity of HDAC2 could effectively relieve ALF^15^. TNF-α involved “two-hit” theory plays an important role in the process of pyroptosis during ALF^29^. Therefore, we speculate that HDAC2 could regulate pyroptosis during ALF. In order to find out which molecules HDAC2 directly affects, HeLa cells were firstly transfected with plasmid to knock-down or overexpress HDAC2. The bioinformatics analysis for the Venn diagram revealed that 1483 and 5721 genes were differentially altered after knockdown and overexpression of HDAC2. Among these genes, there were 636 genes altered as shown HDAC2 upregulated or downregulated (Fig. 1A). These 636 genes were performed with Gene Ontology (GO) analysis. The genes involved in pyroptosis were not directly regulated by HDAC2 in the 636 genes. However, it was found that cell morphogenesis involved in differentiation for the GO Biological Processes was closely related to the characteristics of pyroptosis that the cell expanded until the cell membrane ruptured, leading to the release of its contents (Fig. 1B and Table 1). 11 genes with high correlation with the process of cell morphogenesis involved in differentiation were clustered. Moreover, studies have demonstrated that autophagy can negatively regulate the activation of inflammasome during pyroptosis^9,10^. In the 11 genes, ULK1 was only autophagy molecule associated with the NLRP3-pyroptosis pathway (Fig. 1C). The results of sequencing revealed that the expression of ULK1 gene was negatively correlated with the expression of HDAC2.

**Table 1:** Metascape.

GO	Category	Term	Count	%	*P*-value
R-HSA-1474244	Reactome Gene Sets	Extracellular matrix organization	31	5.04	−10.3
GO:0000904	GO Biological Processes	Cell morphogenesis involved in differentiation	51	8.29	−10.1
R-HSA-1640170	Reactome Gene Sets	Cell Cycle	41	6.67	−7.08
R-HSA-3247509	Reactome Gene Sets	Chromatin modifying enzymes	24	3.9	−6.74
GO:0001568	GO Biological Processes	Blood vessel development	45	7.32	−6.69
GO:0006325	GO Biological Processes	Chromatin organization	46	7.48	−6.64
GO:0030099	GO Biological Processes	Myeloid cell differentiation	30	4.88	−6.54
GO:0048598	GO Biological Processes	Embryonic morphogenesis	37	6.02	−6.48
R-HSA-350054	Reactome Gene Sets	Notch-HLH transcription pathway	6	0.98	−6.41
GO:0007346	GO Biological Processes	Regulation of mitotic cell cycle	40	6.5	−6.1
GO:0007423	GO Biological Processes	Sensory organ development	34	5.53	−5.94
GO:0060322	GO Biological Processes	Head development	43	6.99	−5.81
GO:0051301	GO Biological Processes	Cell division	36	5.85	−5.79
GO:2000147	GO Biological Processes	Positive regulation of cell motility	34	5.53	−5.65
GO:0051962	GO Biological Processes	Positive regulation of nervous system development	33	5.37	−5.52
R-HSA-3108232	Reactome Gene Sets	SUMO E3 ligases SUMOylate target proteins	17	2.76	−5.35
GO:0000280	GO Biological Processes	Nuclear division	28	4.55	−5.28
GO:0042060	GO Biological Processes	Wound healing	33	5.37	−5.12
GO:1904837	GO Biological Processes	Beta-catenin-TCF complex assembly	7	1.14	−5.09
GO:0007017	GO Biological Processes	Microtubule-based process	40	6.5	−4.99

### HDAC2 regulated L02 cell pyroptosis induced by TNF-α/D-Gal when autophagy gene ULK1 was inhibited

In these experiments, CAY10683 and MRT68921 had been shown to have the inhibition effect on HDAC2 and ULK1 in L02 cells (Fig. S1). In order to clarify the effect of HDAC2 on pyroptosis, HDAC2 inhibitors, downregulated lentiviral vectors and/or upregulated lentiviral vectors were used to knockdown and/or overexpress HDAC2 in the cell pyroptosis model. ULK1, NLRP3 and pyroptosis pathway-related molecules were detected. As shown in Fig. S2, Inhibiton of HDAC2 could inhibit pyroptosis induced TNF-α/D-Gal in L02 cell line. The results of this part were the basis of the entire in vitro experiment: (1) In the cell model, it was demonstrated from the protein level that the change of HDAC2 was opposite to that of ULK1. (2) The cell model was accompanied by occurrence of pyroptosis, which meant that the cell pyroptosis model was successfully constructed. Down-regulating HDAC2 could reduce the degree of pyroptosis. (3) The HDAC2 inhibitor and down/overexpressed lentivirus used in this part regulate the expression of HDAC2 from the chemical and biological levels, respectively. In follow-up experiments, HDAC2 was regulated by the above methods to clarify the effect on ULK1 and cell pyroptosis.

**Fig. 2:**
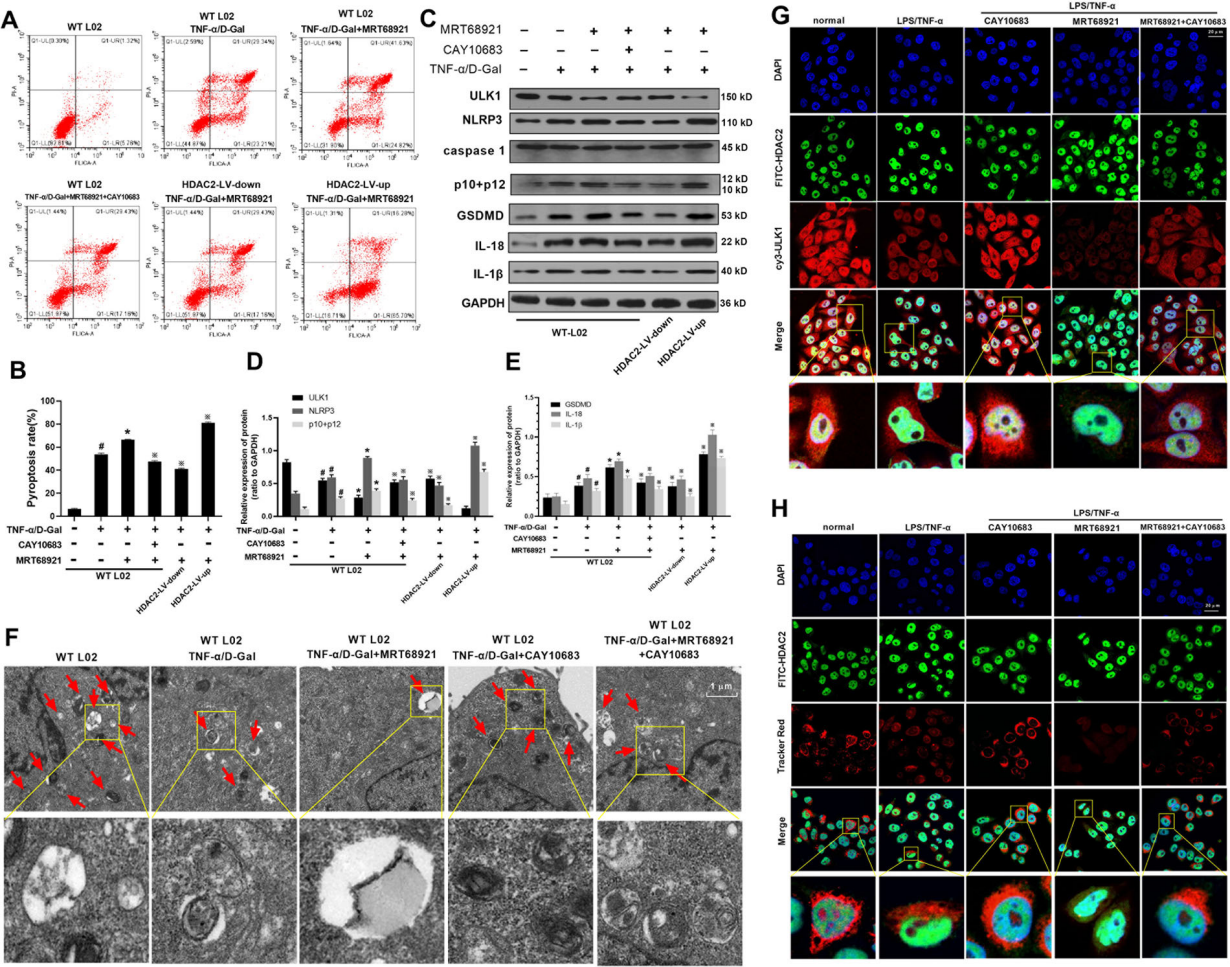
Pyroptosis rate, protein expressions of HDAC2, NLRP3, P10 + P12, GSDMD, IL-18, IL-1β, autophagosome and co-localization of ULK1, HDAC2 and lysosome with HDAC2 interventions and ULK1 inhibitor MRT68921 on TNF-α/D-Gal stimulated hepatocytes. **A**, **B** Compared with WT L02 cell group, the rate of cell pyroptosis was increased in TNF-α/D-Gal group. Compared with TNF-α/D-Gal treated L02 cells, the rate of cell pyroptosis was increased in MRT68921 group. After HDAC2 was downregulated by CAY10683 or by shRNA-HDAC2 lentiviral vector, the rate of cell pyroptosis was decreased compared with MRT68921 group. **C**–**E** The rate of cell pyroptosis was increased after HDAC2 upregulated by lentiviral vector. For the protein expression of ULK1, compared with WT L02 cell group, the protein level of ULK1 was decreased in TNF-α/D-Gal group. Compared with TNF-α/D-Gal group, ULK1 protein level was decreased in MRT68921 group. ULK1 protein level was increased in MRT68921 + CAY10683 or MRT68921 + HDAC2-LV-down group, decreased in MRT68921 + HDAC2-LV-up group compared with MRT68921 group. For the protein expressions of HDAC2, NLRP3, P10 + P12, GSDMD, IL-18, IL-1β, compared with WT L02 cell group, the above protein levels were increased in TNF-α/D-Gal group. Compared with TNF-α/D-Gal group, the above protein expressions were increased in MRT68921 group. The above protein expressions were decreased in MRT68921 + CAY10683 or MRT68921 + HDAC2-LV-down group, increased in MRT68921 + HDAC2-LV-up group compared with MRT68921 group. Compared with WT L02 cell group, the protein levels of HDAC2, NLRP3, P10 + P12, GSDMD, IL-18, IL-1β were increased in TNF-α/D-Gal group. From the above flow cytometry and protein results, it was shown that the regulation of hepatocyte pyroptosis for HDAC2 was an ULK1-dependent pathway. **F** The amounts of autophagosome in TNF-α/D-Gal treated cells were decreased compared with normal cells (WT L02 cells). After being treated with MRT68921 or CAY10683, the amounts of autophagosome were decreased or increased, respectively, compared with TNF-α/D-Gal treated cells. Moreover, when the cells were administrated with both MRT68921 and CAY10683, the amounts of autophagosome were increased (vs. MRT68921 treated cells) or decreased (vs. CAY10683 treated cells), respectively. **G**, **H** The co-localization of ULK1, HDAC2 and lysosome was observed by laser confocal microscopy. Compared with normal L02 cells, the HDAC2 expression was increased in TNF-α/D-Gal treated group. The ULK1 and lysosome expression was decreased. After being treated with CAY10683, the HDAC2 expression was decreased. The ULK1 and lysosome expression was increased. After being treated with MRT68921, there was no change for HDAC2 expression. The ULK1 and lysosome protein expression was decreased, compared with TNF-α/D-Gal treated group. Moreover, when the cells were administrated with both MRT68921 and CAY10683, there was no change for HDAC2 expression, compared with CAY10683 treated cells. ULK1 and lysosome expression was increased. The ULK1 and lysosome expression was decreased or increased, respectively, when compared with CAY10683 treated group or MRT68921 treated group. Data are expressed as the mean ± SEM, and *n* = 3 in each group. ^#^*P* < 0.05, compared with WT L02 cell group. *P < 0.05, compared with TNF-α/D-Gal treated group. ^※^*P* < 0.05, compared with MRT68921 treated group.

In order to clarify how ULK1 regulates this process, ULK1 inhibitor was used in combination with HDAC2 intervention. As shown in Fig. 2A, B, compared with WT L02 cell group, the rate of cell pyroptosis was increased in TNF-α/D-Gal group. Compared with TNF-α/D-Gal treated L02 cells, the rate of cell pyroptosis was increased in MRT68921 group. After HDAC2 was downregulated by CAY10683 or by shRNA-HDAC2 lentiviral vector, the rate of cell pyroptosis was decreased compared with MRT68921 group. Moreover, the rate of cell pyroptosis was increased after HDAC2 upregulated by lentiviral vector.

For the protein expression of ULK1, as shown in Fig. 2C–E, compared with WT L02 cell group, the protein level of ULK1 was decreased in TNF-α/D-Gal group. Compared with TNF-α/D-Gal group, ULK1 protein level was furtherly decreased in MRT68921 group. However, ULK1 protein level was increased in MRT68921 + CAY10683 or MRT68921 + HDAC2-LV-down group, decreased in MRT68921 + HDAC2-LV-up group, compared with MRT68921 group.

The protein expressions of HDAC2, NLRP3, P10 + P12, GSDMD, IL-18, IL-1β were increased in TNF-α/D-Gal group, compared with WT L02 cell group (Fig. 2C–E). Compared with TNF-α/D-Gal group, the above protein expressions were increased in MRT68921 group. However, the above protein expressions were decreased in MRT68921 + CAY10683 or MRT68921 + HDAC2-LV-down group, increased in MRT68921 + HDAC2-LV-up group, compared with MRT68921 group. The protein levels of HDAC2, NLRP3, P10 + P12, GSDMD, IL-18, IL-1β were increased in TNF-α/D-Gal group, compared with WT L02 cell group.

From the above flow cytometry and protein expression results, they were shown that the regulation of hepatocyte pyroptosis for HDAC2 was an ULK1-dependent pathway. Similarly, as shown in Fig. S3, HDAC2 regulated IL-1β secretion in TNF-α/D-Gal induced L02 cell pyroptosis, when ULK1 was inhibited. However, autophagy induction gene ULK1 exerted its biological function through autophagy pathway or non-autophagy pathway^30^. This study explored the role of ULK1 in the autophagy pathway. Therefore, the autophagosome of cells was observed by electron microscope. The amounts of autophagosome in TNF-α/D-Gal treated cells were decreased compared with normal cells (WT L02 cells). When compared with TNF-α/D-Gal treated group, the amounts of autophagosome were decreased in MRT68921 group, increased in CAY10683 group. Moreover, when the cells were administrated with both MRT68921 and CAY10683, the amounts of autophagosome were increased (vs. MRT68921 treated cells) or decreased (vs. CAY10683 treated cells), respectively (Fig. 2F).

When the mitochondria (or other organelles) in the cell were damaged, autophagosomes wrapped in a double-layer membrane were formed, which then fused with lysosomes and were finally degraded^11^. The co-localization of ULK1 and lysosome illustrated the activity of autophagolysosome by laser confocal microscopy. The co-localization of HDAC2 and ULK1 was to observe the regulatory effect of HDAC2 on ULK1 more intuitively. HDAC2 was mainly expressed in cell nucleus. ULK1 and lysosome were mainly expressed in cytoplasm. Compared with normal L02 cells, the HDAC2 expression was increased in TNF-α/D-Gal treated group. The ULK1 and lysosome expression was decreased. After being treated with CAY10683, the HDAC2 expression was decreased. The ULK1 and lysosome expression was increased. After being treated with MRT68921, there was no change for HDAC2 expression. The ULK1 and lysosome protein expression was decreased, compared with TNF-α/D-Gal treated group. Moreover, when the cells were administrated with both MRT68921 and CAY10683, there was no change for HDAC2 expression, compared with CAY10683 treated cells. ULK1 and lysosome expression was increased. The ULK1 and lysosome expression was decreased or increased, respectively, when compared with CAY10683 treated group or MRT68921 treated group (Fig. 2G, H).

The above results indicate that in the process of hepatocyte pyroptosis, the amount of ULK1-mediated autophagosome and lysosome fusion was reduced. Inhibition of HDAC2 could promote the expression of ULK1, promote the formation of autophagic lysosomes, clear the damaged organelles in the cell, and restore the normal function of hepatocytes.

Moreover, as shown in Figs. S4–S5, HDAC2 could regulate L02 cell pyroptosis induced by TNF-α/D-Gal, when pyroptosis was inhibited. As shown in Figs. S6–S8, pyroptosis inhibitor AC-YVAD-CMK, ULK1 inhibitor MRT68921, intervention of HDAC2 were together combined to use in cell model. It was observed that HDAC2 and ULK1 could furtherly regulate pyroptosis in L02 cell model, when AC-YVAD-CMK was used. This cell experimental part showed that HDAC2 could affect pyroptosis through ULK1 autophagy-dependent pathway. This part has an important significance in treating liver injury from the perspective of cell pyroptosis: (1) It provides a scientific basis for clinical trials or in vivo experiments to use pyroptosis inhibitors to treat liver cell injury. (2) In liver cell injury, ULK1-dependent autophagy pathway can affect cell pyroptosis. (2) The pyroptosis inhibitor AC-YVAD-CMK enhances the efficacy of CAY10683 against hepatocyte pyroptosis.

### HDAC2 deacetylated ULK1 by lysine K68 site

**Fig. 3:**
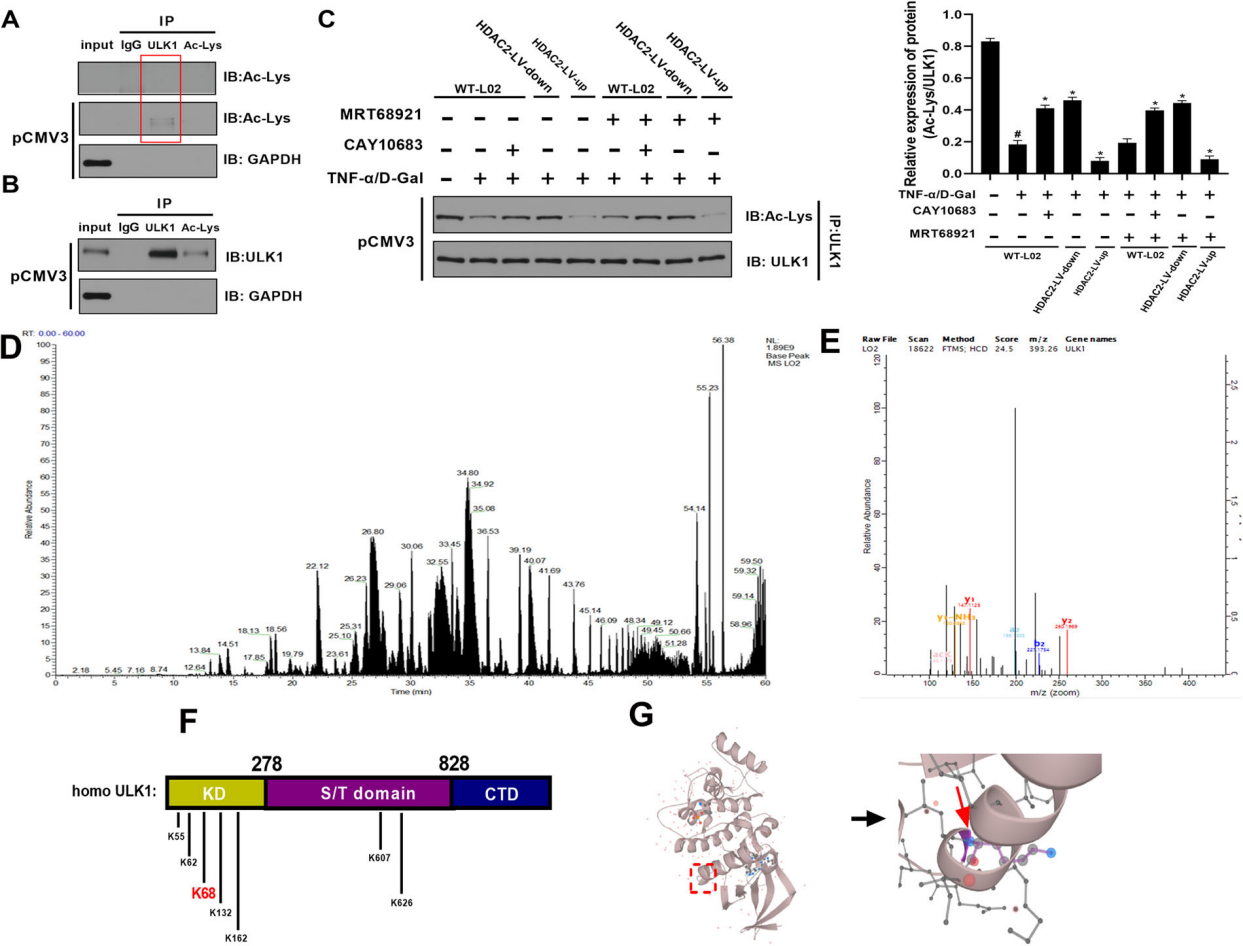
HDAC2 deacetylating ULK1 by lysine K68 site. **A** Total protein in L02 cells was directly extract, then performed with IP for ULK1 protein and Ac-Lys protein. The IB experiment with Ac-Lys protein was then perform. It could be found that ULK1 and Ac-Lys were not bound. However, after L02 cell was transfected with ULK1 overexpression pCMV3 vector, the amounts of Ac-Lys combined with ULK1was obviously increased. **B** After L02 cell was transfected with ULK1 overexpression pCMV3 vector, the amounts of ULK1 combined with Ac-Lys or ULK1 was obviously observed. **C** After interfering with L02 cells in different ways, the protein expression of ULK1 should have been different. However, in order to control the variables, we adjusted the expression of ULK1 by homogenizing the gray value of ULK1 in the western blot gels, and observed the effect of HDAC2 on the acetylated lysine on ULK1 protein under the same expression of ULK1. Compared with WT L02 cells group, the amounts of acetylated lysine combined with ULK1 was decreased. When HDAC2 was downregulated by CAY10683 or shRNA-HDAC2 lentiviral vector, the amounts of acetylated lysine combined with ULK1 were increased. When HDAC2 was upregulated by HDAC2-plasmid lentiviral vector, the amounts of acetylated lysine combined with ULK1 were decreased. During the process, MRT68921 could decrease the amounts of acetylated lysine combined with ULK1. **D** Then Basepeak of IP/MS qualitative results showed peptide signal strength. **E** The secondary mass spectrometry displayed ULK1 K68 lysine site was modified by acetylation in L02 cells (the pink words “acK”). **F** The lysine site of K65, K62, K68, K132, and K162 in kinase domain (KD) area, K806 and K625 in serine/threonine (S/T) domain area were the common acetylation sites for human ULK1 protein. **G** The space structures of K68 lysine site (in the red box) in human ULK1 protein. The right side is a partial enlarged view of K68 lysine site. Ac-Lys: acetylated lysine.

The above results only indicated that ULK1 was regulated by HDAC2 in the process of hepatocyte pyroptosis, but the specific regulation manner was still unknown. Firstly, we proved that ULK1 was a non-histone protein that could bind to acetylated lysine (Ac-Lys). Total protein in L02 cells was directly extract, then performed with IP for ULK1 protein and Ac-Lys protein. The immunoblotting (IB) experiment with Ac-Lys protein was then performed. It could be found that ULK1 and Ac-Lys were not bound. However, after L02 cell was transfected with ULK1 overexpression pCMV3 vector, the amounts of Ac-Lys combined with ULK1 was obviously increased. It was deserved that the amounts of Ac-Lys (IB) combined with Ac-Lys (IP) was still not observed (Fig. 3A). We speculated that on the one hand, the expression of proteins in the acetylated state of normal cells was inherently low, and there was less Ac-Lys bound to these proteins. On the other hand, it might be that the amounts of total protein loaded were not enough to detect Ac-Lys, although the loading of total protein was close to the critical value that SDS gel could withstand. After L02 cell was transfected with ULK1 overexpression pCMV3 vector, the amounts of ULK1 (IB) combined with Ac-Lys (IP) or ULK1 (IP) were obviously observed (Fig. 3B). Secondly, we confirmed that Ac-Lys on the non-histone protein ULK1 could be regulated by HDAC2. After interfering with L02 cells in different ways, the protein expression of ULK1 should have been different. However, in order to control the variables, we adjusted the expression of ULK1 by homogenizing the gray value of ULK1 in the western blot gels, and observed the effect of HDAC2 on the acetylated lysine for ULK1 protein under the same expression of ULK1. Compared with WT L02 cells group, the amounts of Ac-Lys (IB) combined with ULK1 (IP) were decreased. When HDAC2 was downregulated by CAY10683 or shRNA-HDAC2 lentiviral vector, the amounts of acetylated lysine combined with ULK1 were increased. When HDAC2 was upregulated by HDAC2-plasmid lentiviral vector, the amounts of acetylated lysine combined with ULK1 were decreased. During the process, MRT68921 could decrease the amounts of acetylated lysine combined with ULK1 (Fig. 3C). Thirdly, we identified the lysine site on the non-histone protein ULK1 that was regulated by acetylation. Then Basepeak of IP/MS qualitative results showed peptide signal strength (Fig. 3D). The secondary mass spectrometry displayed ULK1 K68 lysine site was modified by acetylation in L02 cells (the pink words “acK”) (Fig. 3E). The details of K68 site is shown in the identification table of acetylation site in Supplementary materials 1. As shown in the identification table, there was only Acetyl (K) in examined amino acid sequences. The sequence window was “LAKSQTLLGKEIKILKELKHENIVALYDFQE”. Moreover, by comparing with ULK1 amino acid sequence in UniProtKB protein database, the first “K” in “ILK(1)ELK” was the modified K68 site. The lysine site of K65, K62, K68, K132 and K162 in kinase domain (KD) area, K806 and K625 in serine/threonine (S/T) domain area were the common acetylation sites for human ULK1 protein (Fig. 3F). The space structures of K68 lysine site (in the red box) in human ULK1 protein. The right side is a partial enlarged view of K68 lysine site (Fig. 3G). The above CO-IP and IP/MS qualitative results showed that ULK1 K68 lysine site was modified by acetylation in L02 cells. Then, we mutated the acetylated lysine K68 site on ULK1 protein to detect whether HDAC2 could affect hepatocyte pyroptosis by modifying K68 lysine site on non-histone ULK1. As shown in Fig. S9, HDAC2 could change of pyroptosis rate and Ac-Lys combined on ULK1 after the acetylated lysine K68 site being mutated.

### The expression of HDAC2 and ULK1-NLRP3-pyroptosis pathway in LPS/D-Gal induced mice ALF model

**Fig. 4:**
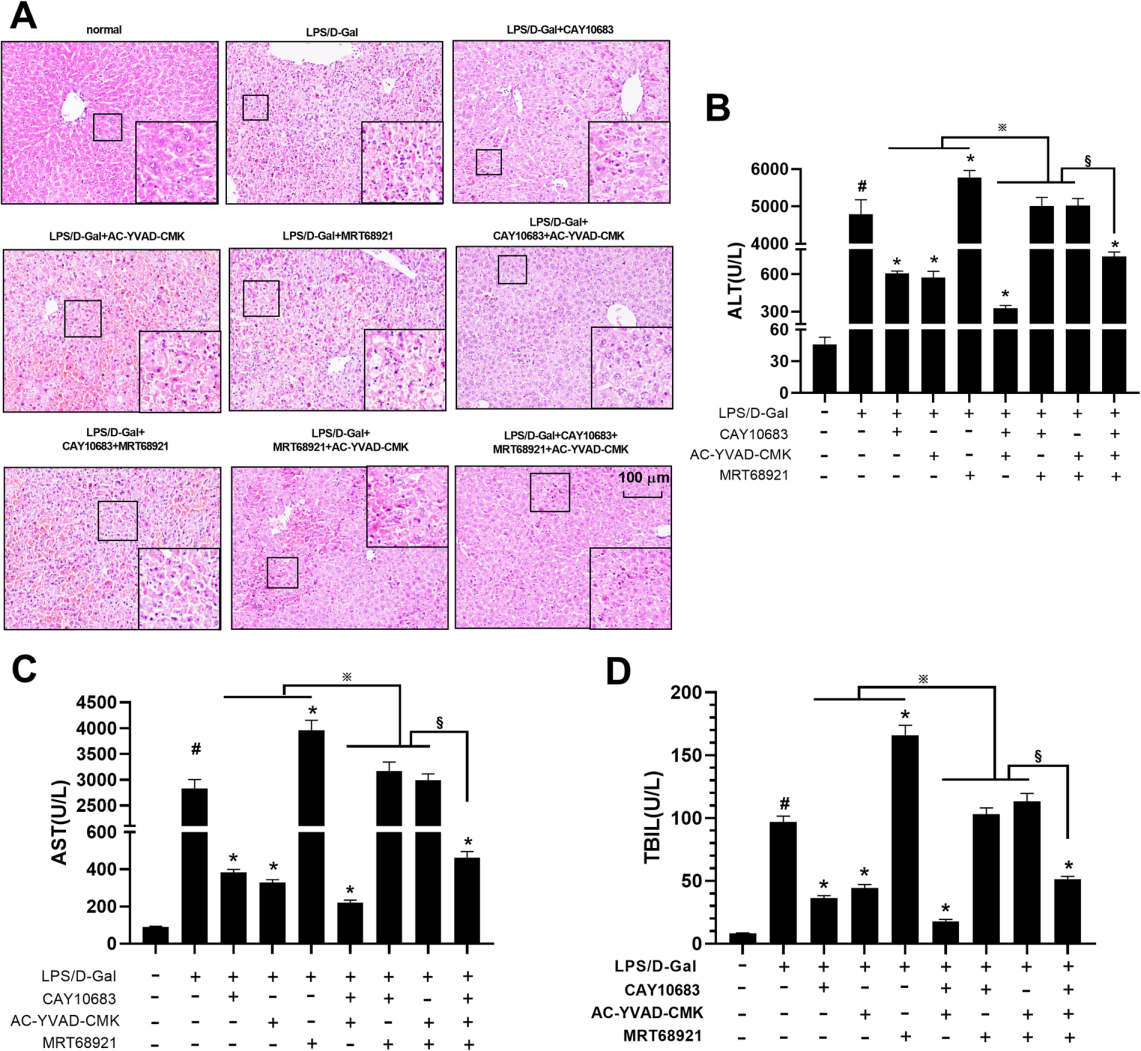
Liver pathologic change, liver function, and cytokines with pyroptosis inhibitor AC-YVAD-CMK, ULK1 inhibitor MRT68921 and HDAC2 inhibitor CAY10683 in ALF mouse model. **A** HE staining revealed neatly arranged hepatocytes in normal liver tissue with no inflammatory cell infiltration. However, hepatocytes in the ALF tissue had a lot of necrosis and inflammatory cell infiltration, and the tissue also contains accumulated red blood cells. CAY10683 and AC-YVAD-CMK could reduce the degree of liver injury, when compared with LPS/D-Gal group. MRT68921 could aggravate liver injury. CAY10683 combined with MRT68921 could play the synergistic effect for liver protecting. However, CAY10683 and AC-YVAD-CMK could play the antagonism effect with MRT68921 in the liver protection. **B**–**D** The levels of ALT, AST, and TBIL in serum were increased in LPS/D-Gal group, when compared with normal group. The levels of ALT, AST, and TBIL were decreased in CAY10683, AC-YVAD-CMK, CAY10683 + AC-YVAD-CMK, CAY10683 + AC-YVAD-CMK + MRT68921 and group, but increased in MRT68921 group, when compared with LPS/D-Gal group. However, when respectively compared with CAY10683, AC-YVAD-CMK, or MRT68921 group, the levels of ALT, AST, and TBIL in CAY10683 + AC-YVAD-CMK, CAY10683 + MRT68921, AC-YVAD-CMK + MRT68921 were respectively decreased, increased, or decreased. The level of ALT, AST, and TBIL in CAY10683 + AC-YVAD-CMK + MRT68921 group was increased (versus CAY10683 + AC-YVAD-CMK group), decreased (versus CAY10683 + MRT68921 group), or decreased (versus AC-YVAD-CMK + MRT68921), respectively. Data are expressed as the mean ± SEM, and *n* = 8 in each group. ^#^*P* < 0.05, compared with normal group. *P < 0.05, compared with LPS/D-Gal treated group. ^※^ and ^§^*P* < 0.05.

In vitro experiments, it was demonstrated that inhibition of HDAC2 could reduce pyroptosis through modulating the K68 lysine site of ULK1. In the following animal experiments, pyroptosis inhibitor AC-YVAD-CMK and ULK1 inhibitor MRT68921 and HDAC2 inhibitor CAY10683 were used to observe the changes of pyroptosis levels and related molecules in ALF mouse model, respectively. HE staining revealed neatly arranged hepatocytes in normal liver tissue with no inflammatory cell infiltration. However, hepatocytes in the ALF tissue had a lot of necrosis and inflammatory cells infiltration, and the tissues also contain accumulated red blood cells. CAY10683 and AC-YVAD-CMK could reduce the degree of liver injury, when compared with LPS/D-Gal group. MRT68921 could aggravate liver injury. CAY10683 combined with MRT68921 could play the synergistic effect for liver protection. However, CAY10683 and AC-YVAD-CMK could play the antagonism effect with MRT68921 in the liver protection (Fig. 4A).

**Fig. 5:**
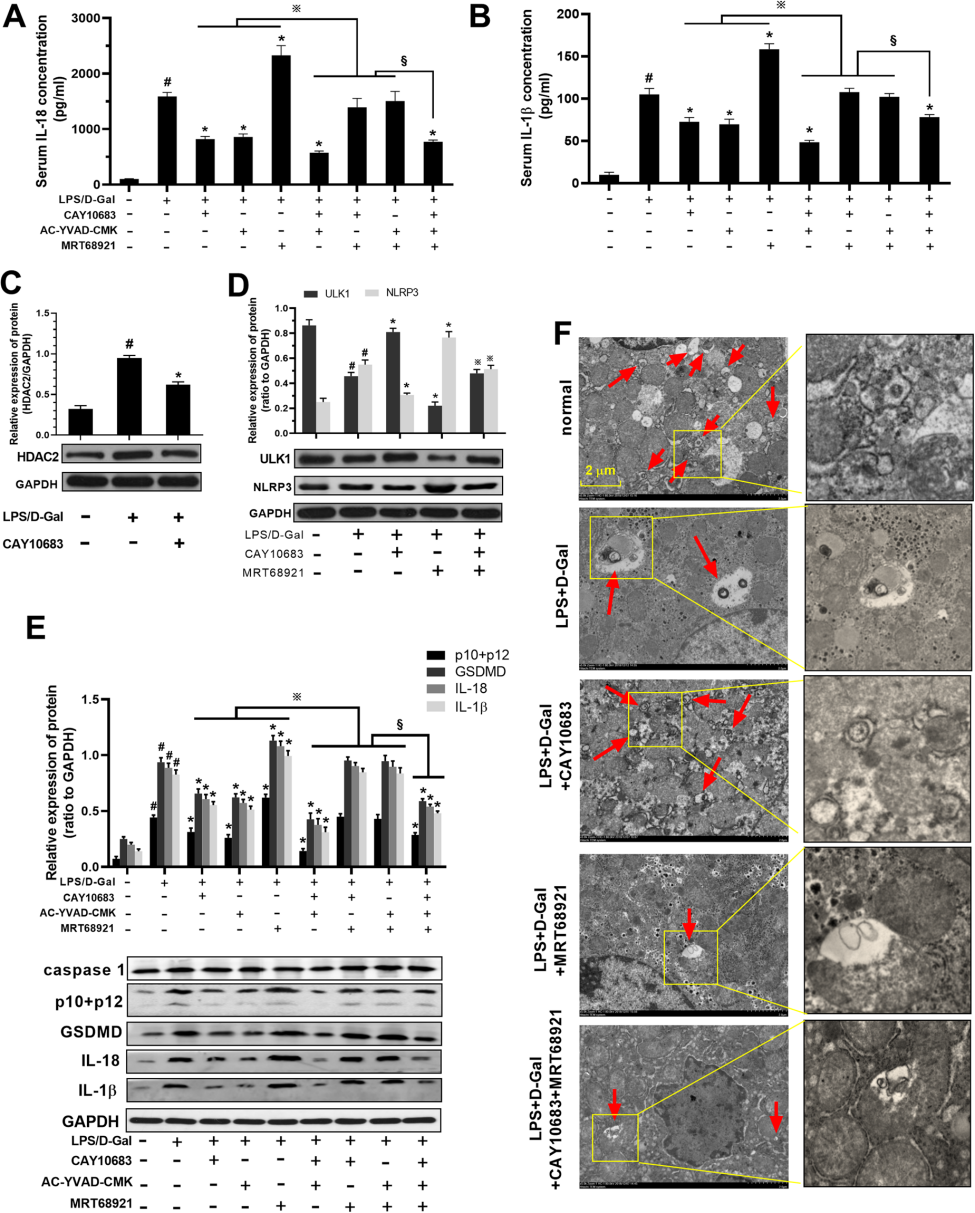
Serum IL-1β, IL-18, protein level of p10 + p12, GSDMD, IL-18, IL-1β, ULK1, NLRP3, and autophagosome in liver with pyroptosis inhibitor AC-YVAD-CMK, ULK1 inhibitor MRT68921 and HDAC2 inhibitor CAY10683 in ALF mouse model. **A**, **B** The serum level of IL-18, IL-1β was detected, and the change trend of IL-18 and IL-1β is consistent with that of ALT, AST, and TBIL. **C** Compared with the normal group, the expression of HDAC2 protein was increased in the LPS/D-Gal group. After CAY10683 treatment, the expression of HDAC2 was subsequently downregulated. **D** Compared with the normal group, the expression of ULK1 protein and number of autophagosomes were decreased in the LPS/D-Gal group. After CAY10683 or MRT68921 intervene, the expression of ULK1 protein and number of autophagosomes were increased or decreased. However, when the two inhibitors were used in combination, the expression of ULK1 protein and number of autophagosomes were decreased or increased, compared to using CAY10683 or MRT68921 alone. However, the NLRP3 protein level shoed the opposite trend with ULK1. **E** The protein level of p10 + p12, GSDMD, IL-18, IL-1β was increased in LPS/D-Gal group, when compared with normal group. The l protein level of p10 + p12, GSDMD, IL-18, IL-1β was decreased in CAY10683, AC-YVAD-CMK, CAY10683 + AC-YVAD-CMK, CAY10683 + AC-YVAD-CMK + MRT68921 and group, but increased in MRT68921 group, when compared with LPS/D-Gal group. However, when compared with CAY10683, AC-YVAD-CMK, or MRT68921 group, respectively, the protein level of p10 + p12, GSDMD, IL-18, IL-1β in CAY10683 + AC-YVAD-CMK, CAY10683 + MRT68921, AC-YVAD-CMK + MRT68921 were decreased, increased, or decreased, respectively. Moreover, the protein level of p10 + p12, GSDMD, IL-18, IL-1β was in CAY10683 + AC-YVAD-CMK + MRT68921 group was increased (versus CAY10683 + AC-YVAD-CMK group), decreased (versus CAY10683 + MRT68921 group), or decreased (versus AC-YVAD-CMK + MRT68921), respectively. **F** Compared with the normal group, the numbers of autophagosomes were decreased in the LPS/D-Gal group. The numbers of autophagosomes was increased in CAY10683 group, decreased in MRT68921 group compared with LPS/D-Gal group. However, when the two inhibitors were used in combination, the numbers of autophagosomes were decreased (versus CAY10683 group), or increased (versus MRT68921 group). Data are expressed as the mean ± SEM, and *n* = 8 in each group. ^#^*P* < 0.05, compared with normal group. *P < 0.05, compared with LPS/D-Gal treated group. ^※^*P* < 0.05 in (**D**), compared with MRT68921 group. ^※^ and ^§^*P* < 0.05.

As shown in Fig. 4B–D and Fig. 5A, B, the levels of ALT, AST, TBIL, IL-1β, IL-18 in serum were increased in LPS/D-Gal group, compared with normal group. The levels of ALT, AST, TBIL, IL-1β, IL-18 were decreased in CAY10683, AC-YVAD-CMK, CAY10683 + AC-YVAD-CMK, and CAY10683 + AC-YVAD-CMK + MRT68921 groups, but increased in MRT68921 group, compared with LPS/D-Gal group. However, the levels of ALT, AST, TBIL, IL-1β, and IL-18 in CAY10683 + AC-YVAD-CMK, CAY10683 + MRT68921, AC-YVAD-CMK + MRT68921 were decreased, increased, or decreased, compared with CAY10683, AC-YVAD-CMK, or MRT68921 group, respectively. Moreover, the level of ALT, AST, TBIL, IL-1β, and IL-18 in CAY10683 + AC-YVAD-CMK + MRT68921 group was increased (versus CAY10683 + AC-YVAD-CMK group), decreased (versus CAY10683 + MRT68921 group), or decreased (versus AC-YVAD-CMK + MRT68921), respectively.

Subsequently, the HDAC2 and ULK1 molecules were detected in ALF mice. As shown in Fig. 5C, compared with the normal group, the expression of HDAC2 protein was increased in the LPS/D-Gal group. After CAY10683 treatment, the expression of HDAC2 was subsequently downregulated. This confirmed that CAY10683 could reduce the high expression of HDAC2 in the liver of ALF mice. As shown in Fig. 5D, compared with the normal group, the expression of ULK1 protein was decreased in the LPS/D-Gal group. After CAY10683 or MRT68921 intervene, the expression of ULK1 protein was increased in CAY10683 group, decreased in MRT68921 group, when compared with LPS/D-Gal group. When the two inhibitors (CAY10683 + MRT68921) were used in combination, the expression of ULK1 protein was decreased (versus CAY10683 group) and increased (versus MRT68921 group), respectively. However, the NLRP3 protein level showed the opposite trend with ULK1.

Moreover, as shown in Fig. 5E, the protein level of p10 + p12, GSDMD, IL-18, IL-1β was increased in LPS/D-Gal group, compared with normal group. The protein level of p10 + p12, GSDMD, IL-18, IL-1β was decreased in CAY10683, AC-YVAD-CMK, CAY10683 + AC-YVAD-CMK, and CAY10683 + AC-YVAD-CMK + MRT68921 groups, but increased in MRT68921 group, compared with LPS/D-Gal group, respectively. However, the protein level of p10 + p12, GSDMD, IL-18, IL-1β was decreased in CAY10683 + AC-YVAD-CMK group (versus CAY10683 group), was increased in CAY10683 + MRT68921 group (versus AC-YVAD-CMK group), was decreased in AC-YVAD-CMK + MRT68921 group (versus MRT68921 group). Moreover, the protein level of p10 + p12, GSDMD, IL-18, IL-1β was in CAY10683 + AC-YVAD-CMK + MRT68921 group was increased (versus CAY10683 + AC-YVAD-CMK group), decreased (versus CAY10683 + MRT68921 group), or decreased (AC-YVAD-CMK + MRT68921), respectively.

As shown in Fig. 5F, compared with the normal group, the numbers of autophagosomes were decreased in the LPS/D-Gal group. The numbers of autophagosomes was increased in CAY10683 group, decreased in MRT68921 group compared with LPS/D-Gal group. However, when the two inhibitors were used in combination, the numbers of autophagosomes were decreased (versus CAY10683 group), or increased (versus MRT68921 group).

In the above experiments, HDAC2/ULK1/pyroptosis inhibitors were used alone an in combination, the results of liver pathology, liver function, serum inflammatory factors and NLRP3-pyroptosis pathway in ALF mice had been verified that: (1) HDAC2 inhibitor and pyroptosis inhibitor had therapeutic effects on ALF mice, ULK1 inhibitors could aggravate the ALF of mice; (2) HDAC2 inhibitors and pyroptosis inhibitors have a synergistic effect in the treatment of ALF mice; (3) ULK1 inhibitors had antagonistic effects on both HDAC2 inhibitors and pyroptosis inhibitors in the treatment of ALF.

### The expression of HDAC2 and ULK1-NLRP3-pyroptosis pathway in liver tissues

**Fig. 6:**
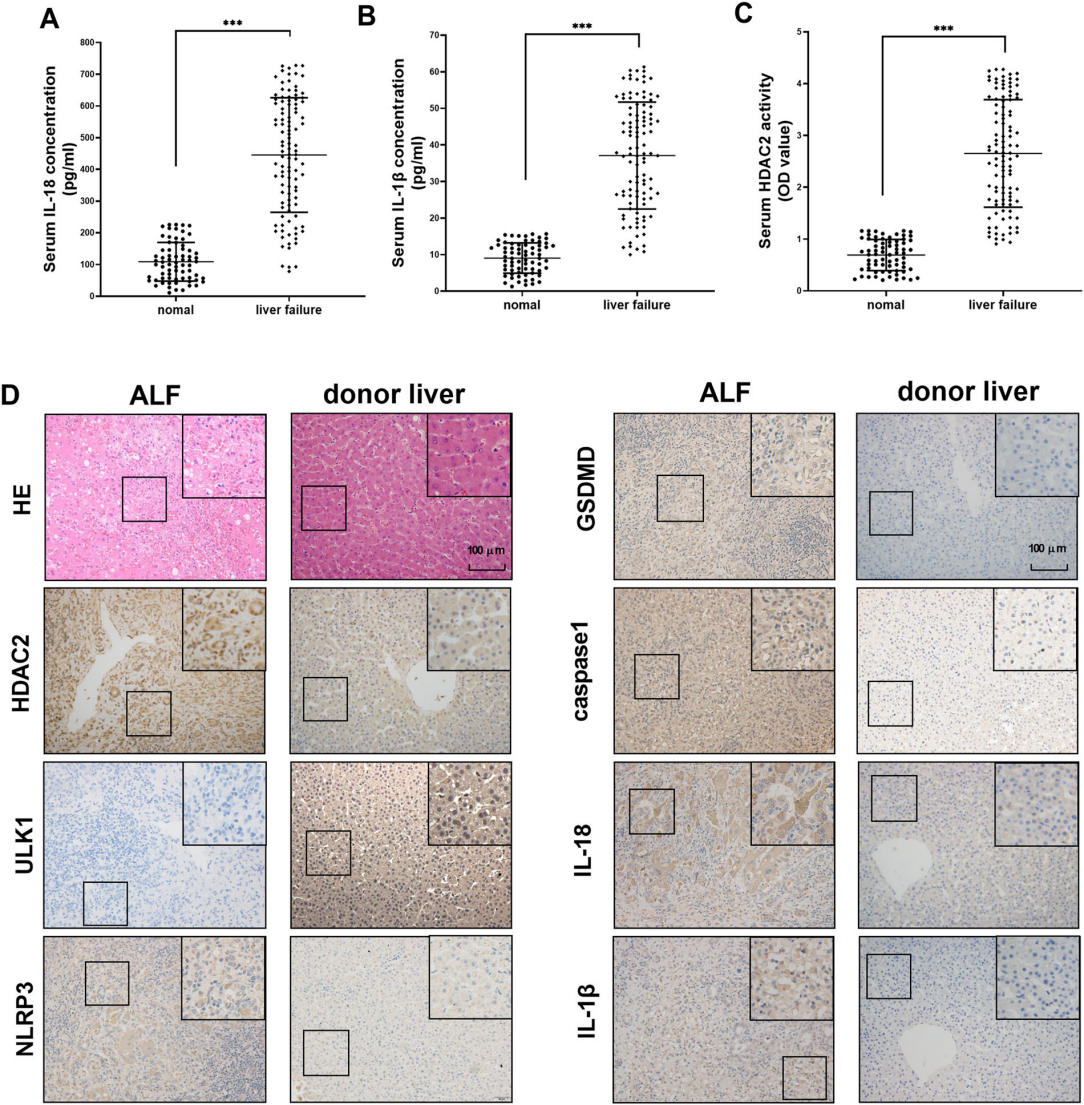
Serum IL-18, IL-1β, HDAC2 level, pathological changes, and protein expression levels of HDAC2, NLRP3, GSDMD, caspase 1, IL-18, and IL-1β in liver tissues of normal people and ALF patients. **A**–**C** The serum IL-18, IL-1β, and HDAC2 level in normal people and ALF patients. The level of HDAC2, IL-18, and IL-1β in liver failure was higher than normal donors. **D** Normal liver tissue showed neatly arranged hepatocytes with no inflammatory cell infiltration. Hepatocytes in the liver failure tissue had a lot of necrosis and inflammatory cell infiltration, and the tissue also contained accumulated red blood cells. The expression levels of HDAC2, NLRP3, GSDMD, caspase 1, IL-18, and IL-1β in ALF patients’ liver tissues were higher than the normal controls. The expression of ULK1 protein expression was reduced. Transmission electron microscopy showed that the nucleus and cell membrane structure were incomplete in the liver failure tissues, and the mitochondria were swollen with a small amount of autophagosomes. Data are expressed as the mean ± SEM. *N* = 70 in normal group, and *n* = 106 in ALF group. ***P < 0.01.

In order to further detect the changes of the above molecules in the ALF patients, 176 subjects (including 70 healthy donors and 106 liver ALF patients) were detected for serum level of HDAC2, IL-18, and IL-1β. The basic information for 176 subjects was shown in Supplementary materials 2. As shown in Fig. 6A–C, the serum level of HDAC2, IL-18, and IL-1β in liver failure patients was higher than healthy donors. The expression of HDAC2 and ULK1-NLRP3-pyroptosis pathway was detected by IHC in health donor and in ALF patients’ liver tissues. As shown in Fig. 6D, normal liver tissue showed neatly arranged hepatocytes with no inflammatory cell infiltration. However, hepatocytes in the liver failure tissues had a lot of necrosis and inflammatory cell infiltration, and the tissue also contained accumulated red blood cells. The expression levels of HDAC2, NLRP3, GSDMD, caspase 1, IL-18, and IL-1β in ALF patients’ liver tissues were higher than the normal controls. However, the expression of ULK1 protein expression was reduced. These results confirmed that HDAC2 and ULK1 had the opposite changes. They regulated together the activation of NLRP3 inflammasome and the pyroptosis pathway in ALF patients.

## Discussion

ALF is a common critical illness in the clinic. Various viral hepatitis, autoimmune liver disease, and drug-induced liver damage are the main causes of ALF. The clinical manifestations of ALF are severe gastrointestinal symptoms, jaundice, and bleeding caused by abnormal coagulation. The recognized pathogenesis of ALF is the “second strike theory”. ALF is often accompanied by intestinal endotoxemia (IETM)^31^. Endotoxin stimulates Kupffer cell proliferation and releases a large amount of TNF-α, which not only directly damages liver cells, but also reduces the ability of the liver to clear endotoxin and aggravate endotoxemia. It, in turn, promotes the production of TNF-α, which aggravates liver damage. Therefore, D-GalN, a sensitizer in liver injury induced by TNF-α^32^, was used as a modeling agent for in vitro cell experiments to mimic the liver injury process^33^. As the definition by EASL or Asian Pacific Association for the Study of the Liver (APASL), ALF is distinct from liver cirrhosis and its mortality is associated with organ failure and systemic inflammation^34,35^. Due to the lacking effective treatment, ALF patient’s conditions are irreversible with the rapid progression, serious complications, complex clinical symptoms, even multiple organ failure^36^. Liver transplantation is the most effective treatment. But the clinical application is limited. There are many disadvantages for the treatment, like multiple postoperative complications, the shortage of donor liver and lifetime immunosuppressant treatment^37^. Therefore, it is a challenge to explore the effective therapies.

Pyroptosis occurs faster than apoptosis and is accompanied by a large release of pro-inflammatory factors^38^. In the classical pathway of pyroptosis, NLRP3 can interact with the pro-caspase-1 binding to the formation of inflammasome. Under the stimulation of inflammatory factors, inflammasome can process pro-caspase-1 into mature caspase-1, thereby promote the maturation of IL-1β and IL-18^5^. During this process, the N-terminal GSDMD protein is highly aggregated and binding lipids. Pores are formed on the cell membrane by combination. The cells gradually rupture with a large release of inflammatory factors IL-18 and IL-1β^6^. The released inflammatory factors can further promote NF-κB signaling pathway to active other immune cells. It replicates inflammatory cascade^39^. All the possible factors that induce liver damage can cause pyroptosis, including hepatotoxic compounds such as benzopyrene (Bap)^40^, CdSe/ZnS quantum dots (QDs)^41^, tunicamycin^42^, LPS^43^, hepatitis C virus (HCV)^44^; surgical injury, such as cecal ligation and puncture (CLP)^45^; physical injury, such as thermal shock^46^. Therefore, pyroptosis may be a good indicator of organ failure and systemic inflammation. Autophagy can negatively regulate the activation of inflammasome, and lysosome/autophagy systems can down-regulate misfolded/aggregated proteins and dysfunctional organelles^9^. As the positive regulator of autophagy, deficiency of MAP1S leads to autophagy defects, which leads to pyroptosis and inflammation^10^. ULK1, the earliest discovered autophagy gene, can promote autophagy by interacting with autophagy-associated protein LC3, and finally inhibit the activity of NLRP3 inflammasomes^11^. If the autophagy-related gene can be upregulated, pyroptosis can be suppressed. “Crosstalk” between autophagy and liver cell pyroptosis causes hepatocyte damage^40^. Therefore, the ULK1-NLRP3-pyroptosis pathway is consistent with the characteristics of liver damage and inflammation during ALF.

The regulation of histone acetylation is accomplished by histone acetyltransferase (HAT) and histone deacetylase (HDAC)^47^. Under normal circumstances, HAT and HDAC protein structure and enzyme activity maintain a high balance. It is called “acetylation homeostasis”. Acetylation regulation plays an important role in maintaining the balance of cell homeostasis. A total of 18 members of HDACs can be divided into four categories^48^: Class I includes HDACs 1, 2, 3, and 8. Class II includes HDACs 4, 5, 6, 7, 9, and 10. Class III includes SIRT 1-SIRT 7. Class IV only includes HDAC 11. The most important role for HDACs is to remove the acetylated modifying groups of histones H2A, H2B, H3, H4, and non-histone^49,50^. HDAC2 is commonly located in nucleus. It is closely related with the process of ALF through regulating intestinal mucosal barrier and intestinal epithelial cell apoptosis^13,14^. In our previous study, HDAC2 can attenuate hepatocyte injury by mitochondrial apoptotic pathway. The specific molecular pathway regulated by HDAC2 was likely to be non-histone^15^. There are few reports on the acetylation-regulated pyroptosis. The PTEN inhibitor can destruct the interaction between SQSTM1 (P62) - HDAC6 protein. The free HDAC6 can inhibit autophagy, which induces apoptosis and pyroptosis^51^.

The acetylation can enhance the expression of inflammatory corpuscle pathway-related genes (TLR2, NLRP3, CD14)^52^. The RNA-seq technology in this study revealed that ULK1 was a negative target regulatory molecule by HDAC2. However, the RNA-seq results were based on the level of transcription of the genes. As a histone/non-histone deacetylation enzyme, the modification of HDAC2 mainly focuses on post-translational modification of molecules. Therefore, we mainly tested whether ULK1 could be regulated by HDAC2 at the protein level. In the cell experiments, targeting knockdown or overexpression of HDAC2 could accordingly up-regulate or down-regulate ULK1, respectively. Then NLRP3-pyroptosis pathway was inhibited or activated, respectively. The ALF mouse induced by D-Gal and LPS was significantly attenuated by HDAC2 inhibitor in mice model, leading to reduced liver tissue pyroptosis and improve liver function. HDAC2 could promote the TNF-α/D-Gal induced pyroptosis cell model or LPS/D-Gal induced mice model, whereas the promote effects was inhibited when autophagy gene ULK1 or pyroptosis was inhibited. During the process of injury, HDAC2 and pyroptosis exerted the antagonistic effect with ULK1. Therefore, HDAC2 in hepatocytes plays a pivotal role in an ULK1-NLRP3 pathway driven auto-amplification of pyroptosis in the inflammation and liver damage of ALF.

We intervened HDAC2, ULK1, and pyroptosis, respectively, but did not interfere with NLRP3. Therefore, we cannot directly judge the effect of NLRP3 on pyroptosis. However, many studies have reported that NLRP3 mediated inflammasome could recognizes a variety of microorganisms, stress, and injury signals. The inflammasomes directly activate caspase 1, then induce the secretion of IL-1β and IL-18, eventually lead to pyroptosis^53–55^. In addition, our study can also indirectly reflect the effect of NLRP3 on pyroptosis. In the cell experiments, it could be concluded that after using HDAC2 inhibitor CAY10683, HDAC2 downregulated lentivirus and HDAC2-upregulated lentivirus, the expression trend of NLRP3 was the same as the key molecules of pyroptosis (GSDMD, caspase 1, IL-1 β, IL-18). The corresponding expression trends were decreased, decreased, and increased. It deserved to note that, the level of pyroptosis was inhibited after the application of pyroptosis inhibitor AC-YYAD-CMK for TNF-α/D-gal stimulated hepatocytes, while the expression of NLRP3 did not change (the results were not shown). This indicated that NLRP3 was the upstream molecules of pyroptosis. On the contrary, HDAC2 could indirectly change the expression of NLRP3, which could cause the change of pyroptosis.

The IP/MS qualitative results showed that ULK1 K68 lysine site was modified by acetylation in L02 cells. It has been reported the lysine site of K65, K62, K132 and K162 in kinase domain (KD) area, K806 and K625 in serine/threonine (S/T) domain area are the common acetylation sites in mouse tissues^56^. However, the reported results only reflect the acetylation site of the ULK1 protein in mice. Our study filled in the blank of humanized ULK1 protein acetylation site modification, which is more in line with the characteristics of clinical precision treatment. Furthermore, the study using ULK1 acetyl-silencing mutants (K68R) showed that HDAC2 could hardly attenuate D-Gal and TNF-α induced pyroptosis in K68R-transfected L02 cells. Although HDAC2 lost its acetylation modification to ULK1 after blocking the K68 site, the pyroptosis viability of the mutant K68 group increased by only about 10% compared to cells that did not knockdown the K68 site (Fig. S9A–S9B). Therefore, we hypothesized that one of the mechanisms that inhibiting HDAC2 to reduce pyroptosis may be due to modulation of the K68 lysine site of ULK1. The RNA-seq results revealed that HDAC2 was a regulator of ULK1. However, this study only discussed the regulation of HDAC2 on the ULK1 protein level. The mechanism of HDAC2 altering UKL1 gene expression at the translational level remained further investigation.

From the clinical study, the high serum levels of HDAC2, IL-18, IL-1β were found in liver failure patients, when compared with normal subjects. The findings might reveal the novel tissues indicators in the pathway of ALF inflammatory cascade. It was consistent with the previous findings that increased levels of pro-inflammatory cytokines have been associated with the severities of inflammation in ALF patients^57^. Meanwhile, this study provides a scientific basis for the clinical application of HDAC2 inhibitors and pyroptosis inhibitors to treat ALF. The K68 lysine site of acetylated non-histone protein ULK1 was used as a target to provide evidence for the diagnosis and treatment of ALF. More important, the K68 lysine site on ULK1 protein was deacetylated by HDAC2 to provide guarantee for the accurate treatment of ALF with HDAC2 inhibitors in the future.

**Fig. 7:**
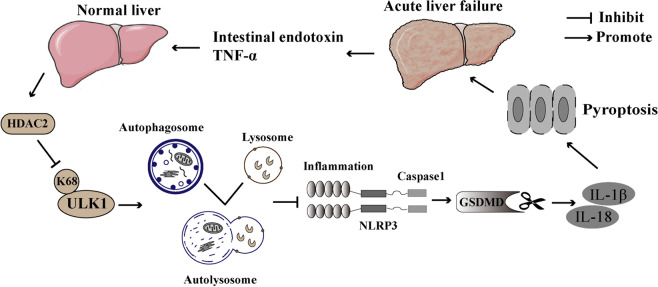
HDAC2 regulating autophagy via ULK1-NLRP3-pyroptosis pathway to influence the inflammatory responses in acute liver failure. In the process of ALF, intestinal-derived LPS promotes the secretion of inflammatory factors, such as TNF-α by inflammatory cells in the liver, and further causes a “two-hit” to the liver. During this process, the level of HDAC2 was increased, and the K68 lysine site on the non-histone protein ULK1 will be deacetylated by HDAC2 to reduce its expression. Then ULK1-dependent autophagosomes activate autolysosomes and further promote the activation of NLRP3 inflammasomes. Inflammasome can process pro-caspase-1 into mature caspase-1, thereby promote the maturation of IL-1β and IL-18. During this process, the N-terminal GSDMD protein was highly aggregated and binding lipids. Pores are formed on the cell membrane by combination. The cells gradually rupture with a large release of inflammatory factors IL-18 and IL-1β. The released IL-1β and IL-18 further aggravate liver damage during ALF. HDAC2 inhibitors and pyroptosis inhibitors showed the treatment effect on ALF. The K68 lysine site of acetylated non-histone protein ULK1 might be the target for the diagnosis and treatment of ALF. More importantly, the K68 lysine site on ULK1 protein was deacetylated by HDAC2 to provide guarantee for the accurate treatment of ALF with HDAC2 inhibitors in the future.

In conclusion, this study illustrates a significant mechanistic finding given the fact that the exacerbation of ALF is thought to be dependent upon the induction of inflammation^58^. The quantification of pro-inflammatory cytokines related to the acetylated ULK1 is useful for management of ALF. Inhibition of HDAC2 reduces pyroptosis by modulating ULK1 acetylation at K68. HDAC2 in hepatocytes plays an important role in an ULK1-NLRP3 pathway driven auto-amplification of pyroptosis in the inflammation of ALF (Fig. 7). However, the early diagnosis and prognostic evaluation value of ALF patients requires further validation of large sample studies and prospective studies. This study would still provide the favorable strategy to design and develop novel drug for treatment of ALF.

## Supplementary information

Supplementary materials 1

Supplementary materials 2
