# Supplementary figures and images for: Histone deacetylase 2 regulates ULK1 mediated pyroptosis during acute liver failure by the K68 acetylation site
Source: Cell Death Dis. 2021 Jan 11;12(1):55. doi: 10.1038/s41419-020-03317-9 (PMC7801742; doi:10.1038/s41419-020-03317-9)

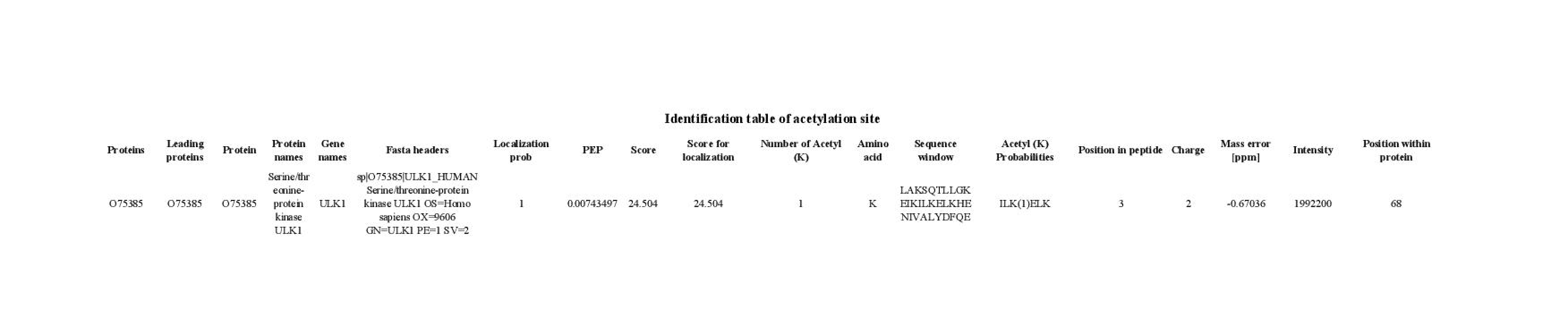

Supplement: Supplementary file 1 — Supplementary materials 1 [file 41419_2020_3317_MOESM1_ESM.tif]

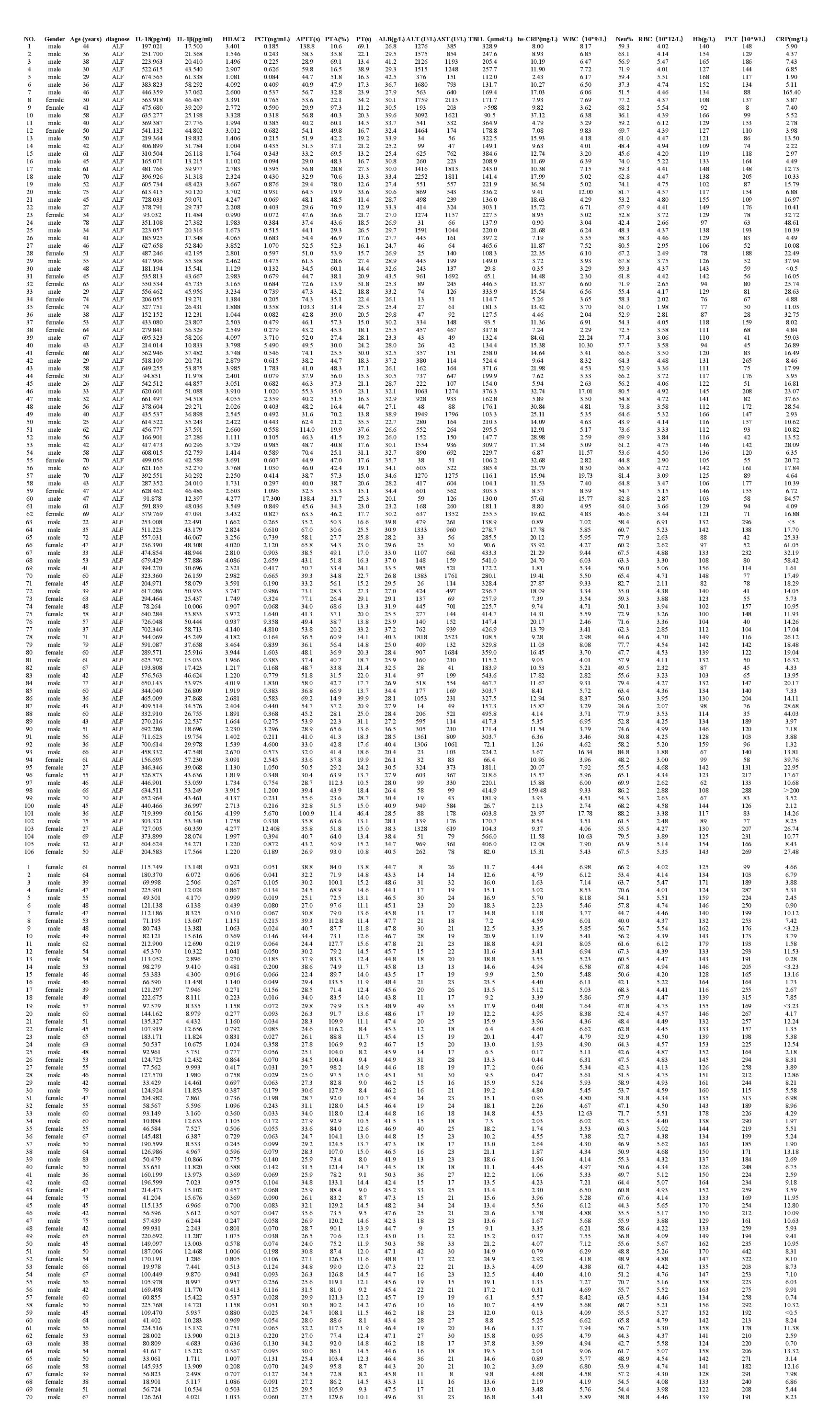

Supplement: Supplementary file 2 — Supplementary materials 2 [file 41419_2020_3317_MOESM2_ESM.tif]
